# EHMTI-0398. Long term safety of the ATI neurostimulation system for the treatment of cluster headache

**DOI:** 10.1186/1129-2377-15-S1-I4

**Published:** 2014-09-18

**Authors:** S Hillerup, J Rostgaard, A Assaf, T Jurgens, M Barlose, M Lainez, O Bigazzi, A Goodman, A Caparso, R Jensen, A May

**Affiliations:** 1 Danish Headache Center, Glostrup Hospital University of Copenhagen, Copenhagen, Denmark; 2 Department of Oral and Maxillofacial Surgery, Rigshospitalet University of Copenhagen, Copenhagen, Denmark; 3 Department of Oral and Maxillofacial Surgery, Universitätsklinikum Hamburg-Eppendorf, Hamburg, Germany; 4 Department of Neurology, Universitätsklinikum Hamburg-Eppendorf, Hamburg, Germany; 5 Department of Neurology, Hospital Clinico Universitario Universidad de Valencia, Valencia, Spain; 6 Clinical Affairs, Autonomic Technologies Inc., Redwood City, USA

## Introduction

At least two-thirds of cluster headache (CH) patients that received the ATI Neurostimulation System have achieved profound clinical improvements including acute headache pain relief and/or significant attack frequency reduction. The ATI Neurostimulator is inserted trans-orally using a minimally invasive technique.

## Aim

This analysis aims to characterize the long term safety of the ATI Neurostimulation System in CH sufferers.

## Method

Patients from the Pathway CH-1 and Pathway R-1 studies were included in the analysis. All adverse events (AEs) including transient swelling and pain, were documented and assessed for relationship to procedure and/or the presence of the neurostimulator.

## Results

Ninety-eight (98) patients received the ATI Neurostimulator as of May 2014. Fifteen patients (15%) reported no AEs and 83 patients (85%) reported at least one related AE. In total, 341 AEs were reported (average 4.1 AEs/patient). Currently, 216 (63%) of all AEs have resolved; average time to resolution was 69 days (range 0-611). The majority of reported AEs (77.4%) occurred within 30 days of the insertion procedure (peri-op AEs). Of these AEs, 82% of patients experienced sensory disturbances. The large majority (72%) of these events had a mild to moderate impact on the patient's daily activities and were transient, with an average resolution of 110.3 days (range 20-313).

## Conclusion

The majority of AEs were reported within 30 days of the Neurostimulator insertion procedure and the majority resolved within 3 months. These AEs are not different from standard sequelae reported for other trans-oral procedures and display a similar time course for healing.

Conflict of interest.

